# Mortality of septic shock patients is associated with impaired mitochondrial oxidative coupling efficiency in lymphocytes: a prospective cohort study

**DOI:** 10.1186/s40635-021-00404-9

**Published:** 2021-07-24

**Authors:** Wagner Luis Nedel, Afonso Kopczynski, Marcelo Salimen Rodolphi, Nathan Ryzewski Strogulski, Marco De Bastiani, Tiago Hermes Maeso Montes, Jose Abruzzi Jr, Antonio Galina, Tamas L. Horvath, Luis Valmor Portela

**Affiliations:** 1 Laboratory of Neurotrauma and Biomarkers, Departamento de Bioquímica, Programa de Pós-Graduação em Bioquímica, ICBS, Universidade Federal do Rio Grande do Sul, Rua Ramiro Barcelos, 2600, anexo, Porto Alegre, RS Brazil; 2 Intensive Care Unit, Hospital Nossa Senhora da Conceição, Grupo Hospitalar Conceição, Porto Alegre, RS Brazil; 3 Zimmer Lab, Departamento de Bioquímica, Universidade Federal do Rio Grande do Sul, Porto Alegre, RS Brazil; 4 Laboratory of Bioenergetics and Mitochondrial Physiology, Instituto de Bioquímica Médica Leopoldo de Meis, Universidade Federal do Rio de Janeiro (UFRJ), Rio de Janeiro, RJ Brazil; 5 Program in Integrative Cell Signaling and Neurobiology of Metabolism, Department of Comparative Medicine, Yale University School of Medicine, New Haven, CT USA

**Keywords:** Septic shock, Lymphocytes, Mitochondrial signatures, Mortality, Prognostic biomarker

## Abstract

**Background:**

Septic shock is a life-threatening condition that challenges immune cells to reprogram their mitochondrial metabolism towards to increase ATP synthesis for building an appropriate immunity. This could print metabolic signatures in mitochondria whose association with disease progression and clinical outcomes remain elusive.

**Method:**

This is a single-center prospective cohort study performed in the ICU of one tertiary referral hospital in Brazil. Between November 2017 and July 2018, 90 consecutive patients, aged 18 years or older, admitted to the ICU with septic shock were enrolled. Seventy-five patients had Simplified Acute Physiology Score (SAPS 3) assessed at admission, and Sequential Organ Failure Assessment (SOFA) assessed on the first (D1) and third (D3) days after admission. Mitochondrial respiration linked to complexes I, II, V, and biochemical coupling efficiency (BCE) were assessed at D1 and D3 and Δ (D3–D1) in isolated lymphocytes. Clinical and mitochondrial endpoints were used to dichotomize the survival and death outcomes. Our primary outcome was 6-month mortality, and secondary outcomes were ICU and hospital ward mortality.

**Results:**

The mean SAPS 3 and SOFA scores at septic shock diagnosis were 75.8 (± 12.9) and 8 (± 3) points, respectively. The cumulative ICU, hospital ward, and 6-month mortality were 32 (45%), 43 (57%), and 50 (66%), respectively. At the ICU, non-surviving patients presented elevated arterial lactate (2.8 mmol/L, IQR, 2–4), C-reactive protein (220 mg/L, IQR, 119–284), and capillary refill time (5.5 s, IQR, 3–8). Respiratory rates linked to CII at D1 and D3, and ΔCII were decreased in non-surviving patients. Also, the BCE at D1 and D3 and the ΔBCE discriminated patients who would evolve to death in the ICU, hospital ward, and 6 months after admission. After adjusting for possible confounders, the ΔBCE value but not SOFA scores was independently associated with 6-month mortality (RR 0.38, CI 95% 0.18–0.78; *P = *0.009). At a cut-off of − 0.002, ΔBCE displayed 100% sensitivity and 73% specificity for predicting 6-month mortality

**Conclusions:**

The ΔBCE signature in lymphocytes provided an earlier recognition of septic shock patients in the ICU at risk of long-term deterioration of health status.

**Supplementary Information:**

The online version contains supplementary material available at 10.1186/s40635-021-00404-9.

## Introduction

Sepsis is a disorder that develops as organ disfunction, and remains one of the leading causes of death globally. Although it may benefit from early diagnostic and prompt treatments to minimize mortality, the availability of diagnostic tools to better predict outcomes and support early clinical decisions are still limited. Septic shock, the most severe end of the spectrum of sepsis, represents a life-threatening condition, which requires vasopressor therapy to treat profound circulatory and metabolic abnormalities [1, 2]. Indeed, during septic shock the oxidative metabolism in several tissues may be limited by the oxygen saturation, delivery, and utilization by mitochondria. Therefore, an early antibiotic therapy, fluid resuscitation and vasopressor drugs alone may not overcome the respiratory deficits at the cellular level. This highlights that inherent or acquired mitochondrial defects associated with septic shock may hinder oxygen consumption coupled with ATP synthesis (OXPHOS), independent of standard therapeutic interventions, thereby contributing to mechanisms underlying organ failure and, eventually, to patient’s death [3–5].

Current advances in the understanding of the physiopathology of sepsis have incorporated the concept of an existing crosstalk between immune cells metabolism and immunity [5–9]. The inflammatory course of septic shock challenges the immune cells to enhance their production of antibodies and signaling molecules over time, which relies on an increased energy consumption supported by the mitochondrial metabolic machinery [7, 10]. Based on this, mitochondria of lymphocytes are constantly reprogramming their metabolism, with the increased activity of oxidative complexes reflecting their higher requirements for ATP synthesis by working at high respiratory rates or the downregulation of oxidative complexes reflecting decreased ATP demands or mitochondrial metabolic exhaustion [9]. These unique features suggest that mitochondria might print metabolic signatures in immune cells of septic shock patients whose clinical significance as a biomarker remains elusive [4, 5, 11–14]. Remarkably, the rates of oxygen consumption associated with individual mitochondrial complexes can be profiled in lymphocytes by respirometry protocols allowing to derive a composite of metabolic signatures that may serve as candidate biomarkers of clinical outcomes. Particularly, the BCE is a parameter which scores how efficiently the mitochondrial machinery are synchronized towards ATP-synthesis [15, 16].

Early studies have analyzed OXPHOS metabolism in sepsis, with controversial results mainly regarding the capability of metabolic endpoints to predict organ failure and mortality [4, 5, 11–14, 17]. In general, they focused on sepsis and did not include a large number of patients with septic shock, which reduced the power of clinical associations between organ failure, mortality, the effectiveness of therapies and specific mitochondrial respiratory states. Also, few studies conducted a strategy of two sequential assessments in the ICU setting and following its impact on the short-, mid- and long-term prognosis.

Therefore, there are still gaps to fill regarding mitochondrial metabolic signatures in immune cells as feasible biomarkers for septic shock patients admitted to the ICU. Accordingly, we aimed to investigate mitochondrial bioenergetic signatures in lymphocytes associated with mortality for septic shock.

## Material and methods

### Participants and study design

**Fig. 1:**
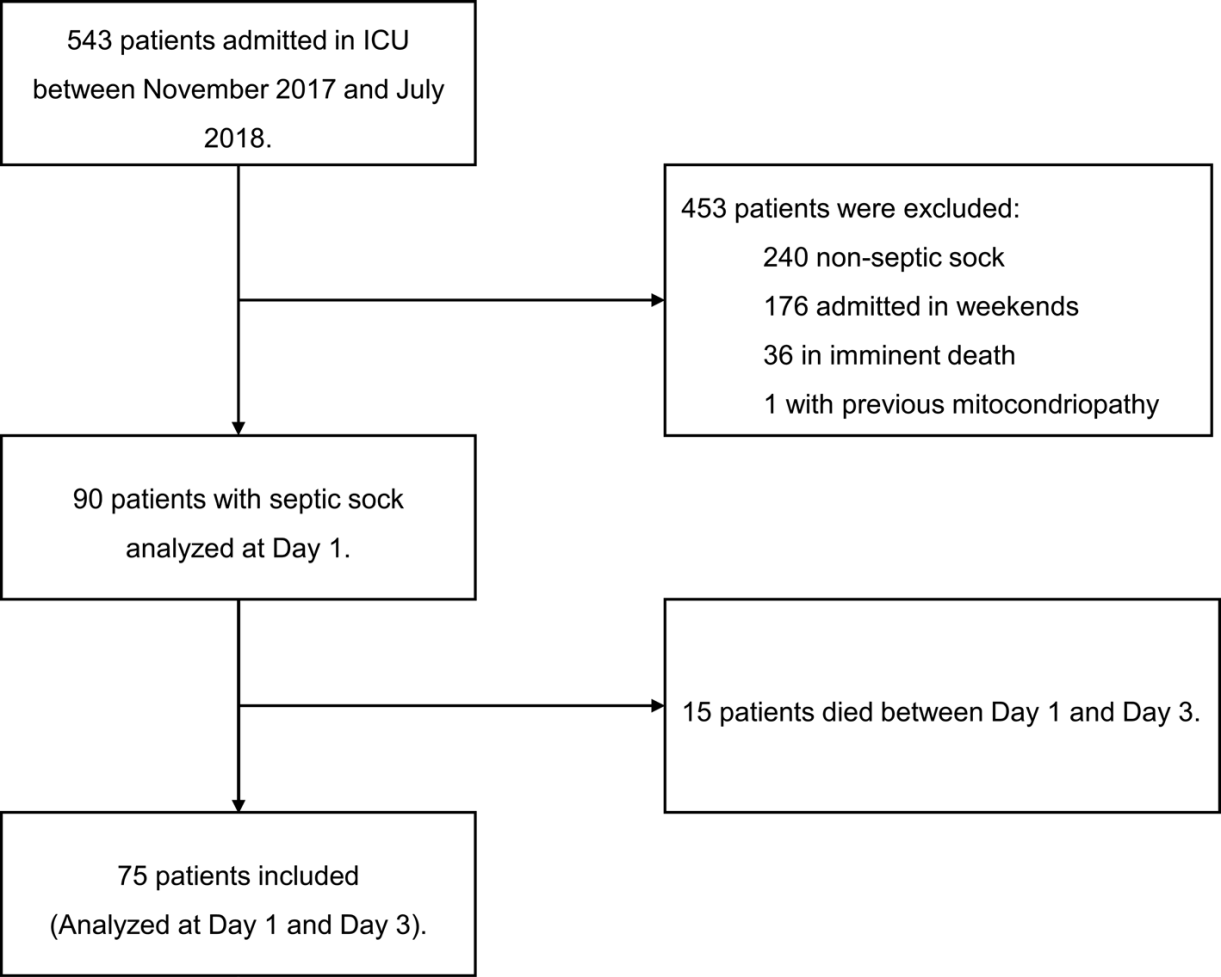
Flow diagram. Ninety patients were initially engaged in the study protocol, but 15 progressed to death on day 1 of admission. Seventy-five septic shock patients completed the study

This is a prospective cohort study designed to evaluate the mitochondrial metabolism of circulating lymphocytes in septic shock patients admitted to four different ICUs from one tertiary university public hospital in Brazil. This study was approved by the local ethics committee (Plataforma Brazil number 66240017.0.0000.5530). We prospectively enrolled 90 adult patients (> 18 years old, 45% were women) admitted to the ICU due to septic shock between November 2017 and July 2018 as indicated in Fig. 1. Patients were excluded if they presented with a known mitochondrial disease, pregnancy, refusal of the patient or the next of kin to sign the informed consent, patients with imminent death, and patients with withholding or withdrawing treatments. Septic shock was defined as the presence of persistent hypotension with the requirement of vasopressor therapy to maintain a mean arterial pressure of 65 mmHg or greater according to Sepsis 2001 definition [18]. The following demographic and clinical characteristics were prospectively recorded: gender, age, primary site of infection, community-acquired or hospital-acquired infection, comorbidities, noradrenaline maximum dose, lactate level, urine output, PaO_2_:FiO_2_ ratio, serum creatinine, total bilirubin, platelets, international normalized rate (INR), Glasgow Coma Scale, capillary refill time, central venous saturation (SvO_2_), length of ICU and length of hospital stay. Also, Simplified Acute Physiology Score (SAPS 3) and the Sequential Organ Failure Assessment (SOFA) score were assessed [19, 20]. The SOFA score was used as a tool for defining both, the clinical condition of the individual patient and the response to therapies [21], as well as delta SOFA as an indicator of resolved or unresolved clinical condition [22]. This score is a gold standard for predicting hospital mortality, providing detailed information regarding the number and severity of organic failure in septic patients [23]. The management of septic shock in these patients was carried out as recommended by Surviving Sepsis Campaign [24] guidelines, especially with regard to early antibiotic therapy, culture collection, fluid replacement and preferential use of norepinephrine as first-line vasopressor drug.

Clinical and laboratory endpoints, including mitochondrial respirometry in permeabilized lymphocytes (digitonin 0.005% w/v), were evaluated at two timepoints: the first day of ICU admission (D1) and three days after admission (D3). The pairwise variations between D1 and D3 (Δ) were used to estimate the improvement or the worsening of the clinical, laboratory and mitochondrial endpoints. SOFA and mitochondrial endpoints were used to dichotomize the survival and death outcomes. The primary outcome was 6-month mortality for septic shock, and the secondary outcomes were ICU and hospital mortality.

### Isolation of lymphocytes and mitochondrial respirometry

Six milliliters of blood were sampled in EDTA tubes at D1 and D3. Lymphocytes were isolated as described by Pecina et al. [25]. We assayed mitochondrial respiration in permeabilized lymphocytes isolated at D1 and D3. The cells counting showed 98% of the lymphocytes in the fraction.

The high-resolution respirometry measurements were performed within 3 h of blood sampling using oxygraphy (Oxygraph-2 k; Oroboros Instruments, Innsbruck, Austria) at 37 °C. Oxygen concentration (micromolar) and oxygen flux (expressed in pmol O_2_.s^−1^.mg of protein) were recorded with DatLab software 6.0. The basal oxygen consumption (oxygen flow per volume or per mass) was established without metabolic substrates. Subsequently, stepwise additions of pyruvate, malate and glutamate (10, 10 and 20 mM, respectively), followed by 2.5 mM ADP, 10 mM succinate, a second 2.5 mM ADP, and finally sodium azide plus antimycin A were performed. This protocol assesses the respiration linked to Complex I (CI), Complex II (CII) and maximal oxygen flow rate consumption coupled to ATP production (P), and nonmitochondrial oxygen consumption [26].

The BCE (also known as ≈ P control factor) was measured to estimate the mitochondrial oxygen flow coupled to ATP production. The BCE is calculated by the P–L/P fluxes; (J ≈ P* = *(P − L)/P), as described previously [15, 16]. A representative image displaying the sequential addition of the substrates to the permeabilized lymphocytes, and the derived respiratory parameters obtained from a control subject, are shown in Additional file 1: Figure S1. The researchers involved in the mitochondrial analysis were blinded to the clinical outcomes, and the researchers involved in the clinical data collection were blinded to the mitochondrial outcomes. All chemicals used for high-resolution respirometry analysis were analytical grade, purchased from Sigma-Aldrich (Sigma-Aldrich, St. Louis, MO, USA).

### Statistical analysis

Descriptive statistics included frequencies and percentages for the categorical variables and means, and standard deviation, confidence intervals, medians, and interquartile ranges for continuous variables. Student’s *t*-test or the Mann–Whitney *U* test were used to compare continuous variables according to normality, assessed by Shapiro–Wilk test. Chi-square test or Fisher’s exact test were used to analyze categorical variables. Association between two continuous variables was measured with Spearman correlation coefficient. To access the impact of BCE improvement at D3 (ΔBCE > 0) on the outcomes, Poisson regression was performed with 6-month mortality as the dependent variable, and hematological neoplasia, chronic kidney disease, lactate at septic shock diagnosis, SOFA improvement at D3 (ΔSOFA < 0) and the SAPS 3 score at ICU admission as independent variables in the model. These variables were selected for the model because they presented a p value of less than 0.20 in the univariate analysis. In this statistical model Δbasal, ΔCI and ΔCII, were not included since they are partial components of the BCE. ΔSOFA score was dichotomized between improvement (ΔSOFA < 0) and worsening (ΔSOFA > 0) on D3, rather than using its value in D1 and D3. The variation between BCE in D3 and D1 (ΔBCE) was analyzed as a continuous variable, in an exploratory analysis. A receiver-operating characteristic (ROC) curve was performed to evaluate the accuracy of ΔBCE to predict 6-month mortality. The optimal cut-off point was mathematically defined using the Youden index [27–31].

Statistical tests were two-tailed with significance defined as a *P* value less than 0.05. All *P* values were two-tailed. We used SPSS version 21.0 (SPSS, Chicago, IL, USA) and R 4.1.0 (R Foundation for Statistical Computing) for all analyses.

## Results

### Clinical and epidemiological characteristics

A total of 90 patients were included in D1, but 15 deceased before D3. A total of 75 patients were included in the D1 and D3 analysis (Fig. 1). The mean age of the patients was 64.8 (± 15.9) years, 42 (56% were male), and 54 (61%) with clinical ICU admission. The most frequent foci of sepsis were the lung (*n* = 41; 46%) and abdominal (*n* = 36; 40%) infections. The mean SAPS 3 score was 75.8 (± 12.9) points and the mean SOFA score at sepsis diagnosis was 8.5 (± 3.2) points (Table 1). At study period, 32% of the patients were submitted to hemodialysis and 85% to mechanical ventilation. Median arterial lactate at septic shock diagnosis was 2.0 mmol/Dl (IQR 1.2–3.0), and the median volume of fluid resuscitation in septic shock was 44 ml/kg (IQR 30–65). Fluid balance in the first day of ICU admission was 2874 ml (IQR 1635–5493). Thirty-seven patients (49%) received hydrocortisone in the septic shock management. Maximum norepinephrine dose in the first day was 0.24 µg/kg/min (IQR 0.07–0.6124 µg/kg/min).

**Table 1:** Patient demographic and clinical variables at admission

Overall population	Distribution and values
Patients, *N*	90
Sex	
Male	50 (55%)
Female	40 (45%)
Age (years)	64.8 (15.9)
Clinical variables	
Surgical patients	35 (38%)
Sepsis foci	
Abdomen	36 (40%)
Cutaneous	2 (2%)
Blood	7 (8%)
Urinary	4 (4%)
Lung	41 (46%)
Capillary refill time (s)	4 (2–6)
Arterial lactate (mmol/L)	2 (1.2–3)
SvO2 (%)	70 (10.2)
PaCO_2_—PvCO_2_ (mmHg)	6.5 (3.9–10.9)
SOFA	8 (3.1)
SAPS 3	75.8 (12.8)
Clinical comorbidities	
Solid cancer	7 (8%)
Blood cancer	7 (8%)
HIV	2 (2%)
Cirrhosis	7 (8%)
Chronic kidney disease	10 (11%)
Diabetes	25 (27%)
Hypertension	32 (35%)

Data are *N* (%), mean (SD), or median (IQR). *SOFA* sequential organ failure assessment, *SAPS 3* simplified acute physiology score 3

### Impact of bioenergetic and clinical variables on mortality

**Fig. 2:**
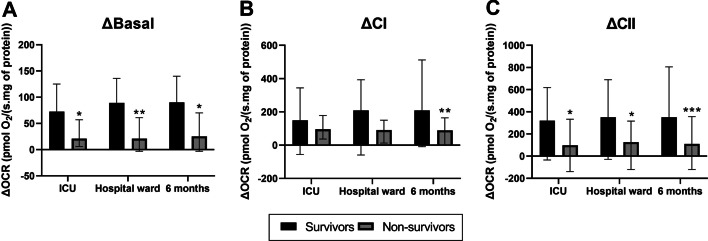
Mitochondrial bioenergetic function is reduced in lymphocytes of non-surviving septic shock patients. Variations (day 3 minus day 1 admission) in mitochondrial oxygen consumption rates in conditions not stimulated by metabolic substrates (**A**, ΔBasal), stimulated by complex I substrates, pyruvate, malate, and glutamate (**B**, ΔCI), and stimulated by complex II substrate, succinate (**C**, ΔCII). Figures are presented as median ± IQR. *Indicates significant differences relative to surviving at *P < *0.05; **P < 0.01; and ***P < 0.001

Overall, mitochondrial metabolic endpoints were associated with short- and long-term mortality. The variations (D3–D1) in the mitochondrial respiration sustained by the presence of endogenous intracellular substrates (ΔBasal), and the stimulated respiration linked to complex II (ΔCII) were significantly decreased in non-surviving patients in all timepoints evaluated (Fig. 2A and C, respectively). Further, stimulated respiration linked to complex I (ΔCI) was decreased in 6 months non-surviving patients relative to surviving (Fig. 2B). Individual values for mitochondrial oxygen consumption at D1 and D3 at basal, linked to CI and CII as well as ΔCII were associated with ICU, hospital and 6-month mortality after sepsis diagnosis (Additional file 3: Table S1). Patients that deceased in the ICU presented elevated arterial lactate, C-reactive protein, capillary refill time and SOFA scores at D1. These patients also had increased SOFA score at and D3 compared with those that survived (Table 2). BCE at D1 and D3 were reduced in non-surviving patients. The number of patients that improved SOFA (ΔSOFA < 0; 11 [34%] versus 38 [88%], *P < *0.001) and BCE (ΔBCE > 0; 2 [6.3%] versus 33 [77%], *P < *0.001) was lower in nonsurvivors (Table 2). Patients that deceased during hospital admission had increased arterial lactate, SOFA at D1 and D3, capillary refill time and SAPS 3. BCE at D1 and D3 are lower in non-surviving than surviving patients. Survivors had greater incidence of improvement in SOFA (ΔSOFA < 0), as well as in BCE (ΔBCE > 0) than nonsurvivors (Table 2).

**Table 2:** Univariate analysis of variables associated with ICU, hospital and 6-month mortality

Variables	ICU		*P*	Hospital		*P*	Six months		*P*
	Survivors *n* = 43	Non-survivors *n* = 32		Survivors *n* = 32	Non-survivors *n* = 43		Survivors *n* = 25	Non-survivors *n* = 50	
Age	64.14 (18.1)	67.16 (14.7)	0.443	61.97 (16.5)	68.00 (16.6)	0.122	62.1 (14.0)	67.1 (17.8)	0.228
Clinical admission at ICU	25 (58%)	21 (66%)	0.51	21 (66%)	25 (58%)	0.51	16 (64%)	30 (60%)	0.737
Sex (male)	26 (60%)	16 (50%)	0.366	18 (57%)	24 (56%)	0.97	15 (60%)	27 (54%)	0.622
Pulmonary sepsis	21 (51%)	12 (38%)	0.24	18 (56%)	16 (37%)	0.101	13 (52%)	21 (42%)	0.412
Solid neoplasia	13 (30%)	4 (11%)	0.07	9 (28%)	8 (18%)	0.33	6 (24%)	11 (22%)	0.845
Hematological neoplasia	2 (4.6%)	3 (9.4%)	0.417	1 (3.1%)	4 (9.3%)	0.386	0 (0%)	5 (10%)	0.162
Cirrhosis	1 (2.3%)	4 (13%)	0.081	1 (3.1%)	4 (9.3%)	0.386	1 (4.0%)	4 (8.0%)	0.66
COPD	7 (16%)	4 (13%)	0.647	5 (16%)	6 (14%)	0.84	3 (12.0%)	8 (16%)	0.742
CKD	5 (12%)	2 (6.2%)	0.428	5 (16%)	2 (4.6%)	0.11	4 (16%)	3 (6%)	0.161
Chronic hypertension	11 (26%)	13 (41%)	0.167	9 (28%)	15 (35%)	0.534	7 (28%)	17 (34%)	0.6
Diabetes	11 (26%)	10 (31%)	0.589	10 (32%)	11 (26%)	0.589	9 (36%)	12 (24%)	0.275
SvO_2_ (%)	68.01 (6.1)	71.36 (11.6)	0.215	67.71 (9.0)	70.70 (11.1)	0.266	68.18 (9.1)	70.06 (10.9)	0.504
Lactate D1 (mmol/L)	1.4 (1.1–2.1)	2.8 (2.0–4.0)	0.002	1.4 (1.1–1.9	2.55 (1.7–3.6	0.015	1.4 (0.9–1.6)	2.2 (1.6–3.5)	0.041
SOFA D1	7.63 (3.0)	1.0 (2.4)	< 0.0001	7.25 (2.55)	9.67 (2.95)	< 0.0001	7.40 (2.6)	9.26 (3.0)	0.011
SOFA D3	3 (2–5)	12.5 (7–19.5)	< 0.0001	3 (1.7–5)	9 (4–16.5)	< 0.0001	3 (1–4)	7.5 (3.25–15.5)	< 0.0001
ΔSOFA < 0	38 (88%)	11 (34%)	< 0.0001	28 (88%)	21 (49%)	< 0.0001	22 (88%)	27 (54%)	0.004
SAPS 3	73.98 (13.2)	78.60 (12.1)	0.1	72.16 (13.1)	78.77 (12.0)	0.026	70.68 (11.2)	78.58 (12.9)	0.011
CRP (mg/L)	153 (69.7–216)	220 (119–284)	0.02	153 (69–223)	196 (119–269)	0.062	153(72.6–209)	188 (98–258)	0.076
CRT D1 (s)	4 (2–4)	5.5 (3–8)	0.04	3.5 (2–4)	4 (3–7)	0.046	4 (3–4)	4 (2.25–7)	0.319
BCE D1	0.354 (0.1)	0.243 (0.1)	< 0.0001	0.378 (0.1)	0.254 (0.1)	< 0.0001	0.380 (0.1)	0.270 (0.1)	< 0.0001
BCE D3	0.456 (0.18)	0.210 (0.1)	< 0.0001	0.535 (0.1)	0.215 (0.1)	< 0.0001	0.543 (0.1)	0.267 (0.2)	< 0.0001
ΔBCE > 0	33 (77%)	2 (6.3%)	< 0.0001	31 (97%)	4 (9.3%)	< 0.0001	24 (96%)	11 (22%)	< 0.0001

Δ indicates the pairwise variation between values obtained at day 3 and day 1, mathematically represented as day 3–day 1 for the indicated variable. Data are mean (SD), median (IQR), or *n* (%).*CRP* C-reactive protein, *SOFA* sequential organ failure assessment, *ICU* intensive care unit, *SAPS 3* simplified acute physiology score 3, *BCE* biochemical coupling efficiency, *COPD* chronic obstructive pulmonary disease, *CKD* chronic kidney disease, *CRT* cardiac resynchronization therapy

Patients who died within 6 months after septic shock presented elevated arterial lactate levels at ICU admission and increased SAPS 3, and had the highest SOFA scores at D1 and D3. Survivors also had greater incidence of improvement in SOFA (ΔSOFA < 0) and in BCE (ΔBCE > 0) than nonsurvivors (Table 2). Remarkably, after adjusting for possible confounders, the 6-month mortality rate was lower in patients who had an improved BCE at D3 (RR 0.38, CI 95% 0.18–0.78; *P < *0.001), while classic laboratory and clinical variables such as lactate, CRP, SOFA and SAPS 3 were not associated with survival or death (Table 3).

**Table 3:** Multivariate analysis of variables associated with 6-month mortality

Variables	Multivariate analysis	*P*
Hematological neoplasia	RR 1.1 (0.37–3.26)	0.86
CKD	RR 0.75 (0.22–2.48)	0.64
Lactate—D1	RR 1.01 (0.88–1.14)	0.88
ΔSOFA < 0	RR 1.01 (0.49–2.08)	0.96
SAPS 3	RR 1.01 (0.98–1.03)	0.321
ΔBCE > 0	RR 0.38 (0.18–0.78)	0.009

Δ mathematically indicates the pairwise variation between values obtained at day 3 (D3) minus day 1 (D1), for the indicated variable. *SOFA* sequential organ failure assessment, *SAPS 3* simplified acute physiology score 3, *BCE* biochemical coupling efficiency, *CKD* chronic kidney disease, *RR* risk ratio

BCE at D1 was inversely associated with SOFA (Spearman = − 0.28; *P = *0.005), but had no associations with arterial lactate (Spearman = − 0.2; *P = *0.057) or with plasma C-reactive protein levels (CRP) (Spearman = 0.007; *P = *0.947). Six patients (8%) developed a need for a tracheostomy during ICU admission. These patients worsened in the ΔBCE when compared to those who did not need a tracheostomy (− 0.085 ± 0.1 versus 0.048 ± 0.14, respectively; *P = *0.11), but without reaching statistical significance. Thirty-six patients developed a new infection after the initial injury, and these patients had no statistically significant difference in their median ΔBCE when compared to those who did not developed reinfection (− 0.008 ± 0.23 versus 0.089 ± 0.19, respectively, *P = *0.29). Patients who needed MV had lower values of ΔBCE when compared with those who did not need it, reaching statistical significance: 0.006 ± 0.14 versus 0.1 ± 0.13, respectively; *P = *0.05. Patients who needed hemodialysis had worsening in the ΔBCE, while those who did not need hemodialysis had an improvement in their values (− 0.029 ± 0.1 versus 0.07 ± 0.2; *P < *0.01). Patients who improved ΔBCE (ΔBCE > 0) had a shorter ICU length of stay when compared with those who impaired ΔBCE (ΔBCE < 0): 6 ± 4 days versus 9.5 ± 14 days, respectively (*P = *0.02). The AUROC for ΔBCE as prognostic biomarker of 6-month mortality was 0.90 (95% CI 0.83–0.91, *P < *0.01) (see Additional file 2: Figure S2). A cut-off point of − 0.002 in ΔBCE had a 100% of sensitivity and 73% of specificity for the outcome (Youden’s J Index = 0.73). Distinct cut-off values of ΔBCE relative to sensitivity and specificity, and Youden’s J Index are shown in Table 4.

**Table 4:** Sensitivity, specificity and Youden’s J index for measurements of ΔBCE

ΔBCE	Sensitivity	Specificity	Youden’s J Index
− 0.220	100%	0%	0
− 0.111	98%	31%	0.31
− 0.002	100%	73%	0.73
0.11	70%	88%	0.58
0.219	33%	97%	0.31
0.354	3%	100%	0.03

## Discussion

Our findings highlight a high short- and long-term mortality among septic shock patients. Mitochondrial metabolism in the lymphocytes of these patients provided a particular signature for the deterioration of clinical outcomes. Among the clinical and mitochondrial metabolic endpoints, ΔBCE was independently associated with long-term mortality in our cohort.

Although SOFA is a well-validated instrument to estimate mortality risk, the addition of new and more sensitive laboratory variables to this score may benefit its predictive validity. Indeed, in the “The Third International Consensus Definitions for Sepsis and Septic Shock (Sepsis-3)” [1], Kramer suggests the incorporation of new candidate biochemical markers to improve the SOFA score sensitivity. Such an approach is pertinent because it may provide earlier information to support clinical decisions, and thus, patients are more likely to achieve better clinical outcomes that reflect their prognosis beyond the ICU [1, 32]. In this context, our approach does not rule out the clinical relevance of SOFA or other clinical scores, but proposes “a look” to a promising complementary prognostic tool. Physiologically, the mitochondria are capable of adjusting their metabolic energy demands and signals to protect cells from insults [7, 9, 14]. This has been clinically investigated in immune cells of patients with sepsis and in septic shock, to estimate the influence of bioenergetics on inflammatory responsiveness, severity of symptoms and the deterioration of health status due to organ failure [9, 33, 34]. Such relevance is conceptually well-illustrated by proposals that activated immune cells can undergo complex plasticity phenomena, which is sustained by mitochondria, “the powerhouses of immunity” [6]. Accordingly, it was demonstrated in blood mononuclear cells that mitochondrial dysfunction and damage progress over time, along with the severity of symptoms, albeit strong conclusions were limited by the small number of patients [6, 9, 34].

Here, we showed an improved metabolism from D1 to D3 (∆BCE) admission to the ICU which paralleled survival, implying that increments in mitochondrial bioenergetic function may reflect metabolic reprograming leading to improvements of the general health status in septic shock patients. Another study found that an early normalization of mitochondrial biogenesis genes expression profile accelerated sepsis recovery and shortened the time in the ICU [34], thus reinforcing the suggestion of clinical and functional relevance in approaching mitochondrial phenotypes. Although we do not address gene-profile, our study conciliates the mechanistic concept that an increased biogenesis governs plasticity mechanisms directed to renew damaged mitochondria, and as consequence, increase energetic efficiency. On the other hand, some intrinsic features support the investigation of mitochondrial bioenergetic effectors in immune cells as biomarkers of outcomes. For instance, dysregulation of immune cells, including lymphocytes, are key components of inflammatory overreaction and organ failure, considered as hallmarks of septic shock; and as resident cells in the circulation, they survey whole body microenvironments, are cells easy to sample and to perform metabolic profiling in routine intensive care practice. The association between improved mitochondrial function with clinically relevant outcomes, such as length of stay in the ICU, the need for mechanical ventilation and the need for hemodialysis, reinforces this hypothesis.

The other clinically relevant spectrum of the sepsis, the compensatory anti-inflammatory response syndrome (CARS), is an event that occurs in a subgroup of septic patients that develop profound acquired immunosuppression. Such condition may cause difficulties to efficiently eradicate the primary infection despite patients are submitted to standardized treatment protocols [35]. Hence, it is important to take into consideration that sepsis leads to a complex immune response that evolves over time, with the simultaneous implication of both, proinflammatory and anti-inflammatory mechanisms for the clinical outcomes [36]. The intensity and duration of the mentioned exacerbated anti-inflammatory phase seem to be closely related with the development of nosocomial infections. Also, an immunometabolic reprogramming in response to sepsis may rely on the interplay between the energy demands and capacity of lymphocyte mitochondria to produce ATP [37]. However, whether or not a metabolic reprograming, here mirrored by BCE, exerts a significant influence in the state of immunosuppression remains to be explored [38]. It is known that the lymphocytes challenged by sepsis displayed an exhausted-like phenotype characterized by decreased functions including the capacity of proliferation, cytokine production and increased coinhibitory receptor expression, culminating in apoptotic cell death [35]. Considering that it is not completely known the exact extrinsic and intrinsic factors that determine the severity of lymphocytes exhaustion, and likely immunosuppression, we tend to suggest decreased mitochondrial bioenergetics as an active player. Accordingly, in physiological conditions lymphocytes are highly oxidative cells which implies in immunological processes highly dependent of an appropriate ATP support. Studies have shown a bidirectional interaction between mitochondria and molecular inflammatory effectors that drive cell responses to an infection. This crosstalk between immunogenic effectors and mitochondrial activity involves toll-like receptors, damage-associated molecular patterns (DAMP’s), pathogen-associated molecular patterns (PAMP’s); and NLRP3 inflammasome activation, all driving mitochondrial mechanisms such as increased reactive oxygen species production, and lymphocyte cytokines release [39, 40].

To expand the exploratory analysis of our study, we found that patients who did not develop a secondary infection, who theoretically would be less susceptible to immunosuppression, had greater increases in BCE when compared to those who had a secondary infection. However, this result was not statistically significant and the sample size was not designed for this analysis, being any conclusions based on these observations merely conjectures that deserve attention in future studies.

Moreover, in cases of unresolved inflammatory-related immunosuppression activity, this condition may progress to a persistent inflammation–immunosuppression and catabolism syndrome (PICS), that often evolve to a chronic critical illness state [41]. A hallmark of this condition is the need for a tracheostomy during the course of septic shock, that is associated with impairment in adaptive immunity related to lymphocytes [42]. In our study, patients who needed a tracheostomy had a worsening in BCE (data no shown), when compared with those who did not. Despite this result did not reach statistical significance, we believe that the small number of tracheostomized patients in our sample could explain this fact.

Our study has the merits of enrolling a large number of septic shock patients in which the respiratory abnormalities in specific mitochondrial complexes represent immunometabolic targets associated with disease pathogenesis. Indeed, no previous study has integrated the activity of mitochondrial complexes CI, CII, and CV, and BCE in lymphocytes using two time-points, which allowed us to estimate metabolic reprogramming relative to the disease progression. The resulting improvement or worsening of the mitochondrial respiratory endpoints in the ICU highlight potential immunometabolic mechanistic targets, and also provide perspective regarding how metabolic reprogramming influences clinical outcomes. This approach outlined BCE as a candidate prognostic biomarker to be incorporated into clinical practice to support therapeutic management and decision-making. Although we provided promising insights regarding mitochondria bioenergetics as biomarker, this approach still requires advances to reach a clinical applicability. The development of ready-to-use assays may increase the potentialities of clinical utilization, nonetheless before pursuing this goal, it is imperative to identify specific mitochondrial functions implicated in the response to the disease.

Our study has a number of limitations that should be further explored. It is a unicentric study, which may limit the external validity of the main findings. Second, it is a population that presented with a high risk of mortality independent of the septic shock. Even so, the mortality reported here reflects the severity measured by the SAPS 3 score, and it is similar to previous studies performed in Brazil and worldwide [43–45]. Third, we evaluated lymphocytes as a whole, albeit different types of lymphocytes may have specific metabolic phenotypes; however, such specificity did not interfere with the predictive nature of ∆BCE. Indeed, the sensitivity and specificity ∆BCE assessed with a ROC curve further confirmed its potential prognostic value for 6- month mortality. As a future perspective, it is important to investigate associations with other clinically relevant outcomes, such as clinical frailty, quality of life, long-term functional status, secondary immunosuppression, incidence of PICS and chronic organ failure after septic shock.

## Conclusions

Our work highlights that impaired mitochondrial capacity to improve BCE provided an earlier recognition of septic shock patients in the ICU at risk of deterioration of health status. On the contrary, improved BCE capacity was associated with favorable clinical outcomes. Further research is needed to outline BCE as a prognostic biomarker to be incorporated into intensive care setting.

## Supplementary Information

**Additional file 1: Figure S1.** Respirometry curve profile. Representative image of a respirometry assay performed in lymphocytes from a control subject.

**Additional file 2: Figure S2.** Receiver-operating characteristic curve. The BCE at a cut-off value of − 0.002 (indicated by the black dot) represents 100% sensitivity and 73 % specificity for predicting 6-month mortality. The obtained area under the curve was 0.90.

**Additional file 3: Table S1.** Mitochondrial respiratory rates in lymphocytes of survival and nonsurvival patients.

## Data Availability

Individual participant data that underlie the results reported in this article, after deidentification, will be made available to researchers who provide a methodologically sound proposal for analyses to achieve aims in the approved proposal, immediately following article publication. Please address the proposals directly to roskaportela@gmail.com.

## Abbreviations

ICU: Intensive care unit
SOFA: Sequential organ failure assessment
SAPS 3: Simplified acute physiology score 3
D1: Day first admission ICU
D3: Day 3 admission ICU
Δ: Values obtained at day 3 minus day 1
CI: Mitochondrial complex I
CII: Mitochondrial complex II
BCE: Biochemical coupling efficiency
IQR: Interquartile range
