# Supplementary material for: Mortality of septic shock patients is associated with impaired mitochondrial oxidative coupling efficiency in lymphocytes: a prospective cohort study
Source: Intensive Care Med Exp. 2021 Jul 24;9:39. doi: 10.1186/s40635-021-00404-9 (PMC8310546; doi:10.1186/s40635-021-00404-9)
Supplement: Supplementary file 1 — Additional file 1: Figure S1. Respirometry curve profile. Representative image of a respirometry assay performed in lymphocytes from a control subject. [file 40635_2021_404_MOESM1_ESM.docx]

**Nedel et al. Supplemental material Representative image of a respirometry assay performed in lymphocytes from a healthy subject.**

**
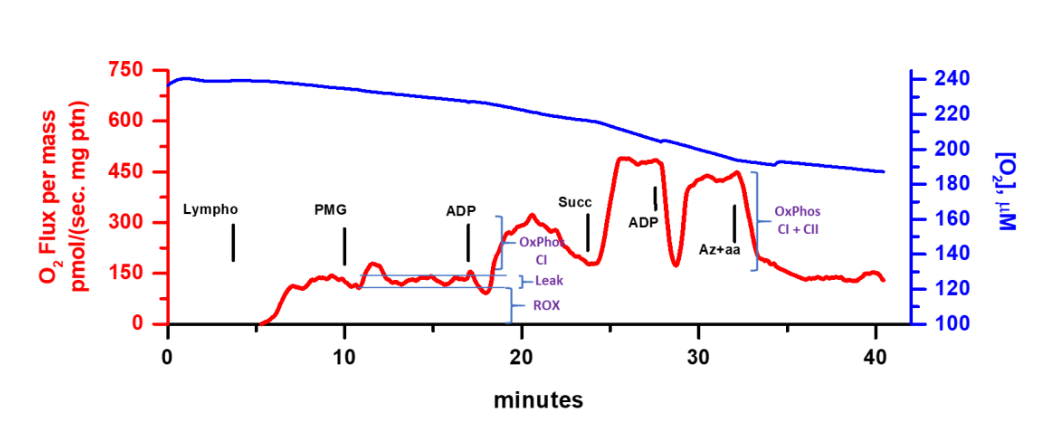
**

**Supplemental Figure 1. Representative image of a respirometry assay performed in lymphocytes from a healthy subject.** After basal respiration was reached, pyruvate, malate and glutamate (10, 10 and 20 mM, respectively) were added, leading to corresponding leak respiration (L), followed by stepwise additions of 2.5 mM ADP, 10 mM succinate, and a second 2.5 mM ADP, allowing for the steady-state rate of oxygen consumption in each step (oxygen flow per volume or per mass). This substrate titration protocol enables derivation of the Complex I (CI), Complex II (CII) and maximal oxygen flow rate consumption coupled to ATP production (State III). Sodium azide plus antimycin A was added to inhibit mitochondrial complex IV, allowing for the estimation of non-OXPHOS oxygen flow rate (ROX) and these fluxes were discounted to obtain an authentic flow rate from all of the abovementioned states, and tissue-mass specific oxygen fluxes were compared in different substrates and coupling states, and inhibitors (SUIT protocol) (ROX). Lymphocytes were permeabilized with 0.005%, digitonin w/v.
