# Supplementary material for: Mortality of septic shock patients is associated with impaired mitochondrial oxidative coupling efficiency in lymphocytes: a prospective cohort study
Source: Intensive Care Med Exp. 2021 Jul 24;9:39. doi: 10.1186/s40635-021-00404-9 (PMC8310546; doi:10.1186/s40635-021-00404-9)
Supplement: Supplementary file 3 — Additional file 3: Table S1. Mitochondrial respiratory rates in lymphocytes of survival and nonsurvival patients. [file 40635_2021_404_MOESM3_ESM.docx]

**Table 2. Mitochondrial respiratory rates in lymphocytes of survival and non-survival patients.**

| Variables­­­­ | ICU | | | Hospital ward | | | Six-months | | |
| --- | --- | --- | --- | --- | --- | --- | --- | --- | --- |
|  | Survivors  n=43 | Non-survivors  n=32 | *P* | Survivors  n=32 | Non-survivors  n=43 | *P* | Survivors  n=25 | Non-survivors  n=50 | *P* |
| Basal D1 | 192.3  (99.7) | 128  (43.5) | <.001 | 208  (104.7) | 131.1  (46.7) | <.001 | 214  (116.9) | 128.7  (43.9) | .001 |
| Basal D3 | 250.4  (88.7) | 157.7  (51.9) | <.001 | 276  (79) | 159  (53.6) | <.001 | 257.7  (51.2) | 149.3  (68.8) | <.001 |
| ΔBasal | 72.3  (3 - 125) | 21.2  (6 - 57) | .04 | 89.1  (24 - 136) | 21.2  (-3 - 61) | .01 | 90  (23 - 140) | 25.3  (-3 - 70) | .03 |
| CI D1 | 445.1  (248.3) | 197.1  (64.3) | <.001 | 503.7  (245.6) | 210.7  (86.1) | <.001 | 525.2  (231.2) | 235.3  (142.5) | <.001 |
| CI D3 | 606.8  (308.5) | 295.4  (92.6) | <.001 | 697.6  (285.9) | 294.8  (100.5) | <.001 | 756.2  (386.5) | 296.5  (146.9) | <.001 |
| Δ CI | 149.5  (-56 - 344) | 95.7  (37 - 178) | .4 | 209.2  (-58.7 - 393) | 90.9  (12.8 – 150) | .07 | 209.2  (-9 - 512) | 88.9  (-1 - 164) | .01 |
| CII D1 | 698.2  (348.2) | 498.4  (182.2) | .01 | 760.2  (355.5) | 494.1  (191.8) | <.001 | 715.9  (309.4) | 555.2  (297.6) | .02 |
| CII D3 | 1026.9  (481.3) | 577.8  (193.2) | <.001 | 1138.8  (466.7) | 596.4  (230.1) | <.001 | 1174.5  (513.7) | 671.7  (296.2) | <.001 |
| ΔCII | 319  (-35 - 618) | 98.5  (-140 to 333) | .03 | 350.6  (-29 - 689) | 126.4  (-121 - 316) | .02 | 350.6  (32 – 805) | 110.9  (-121 - 356) | <.001 |

Basal reflects respiratory rates without addition of exogenous substrates; D1: day first admission ICU; D3: day 3 admission ICU; CI: mitochondrial complex I; CII: mitochondrial complex II. Δ mathematically indicates the pairwise variation between values obtained at day 3 minus day 1, for the indicated variable. Respiratory rates of CI, CII, and the equivalent Δ values are expressed as pmol O_2_ ·s^-1^ ·mg of protein. Data are mean (SD) or median (IQR). Lymphocytes were permeabilized with digitonin 0.005% (w/v).
